# The Efficacy and Safety of Fecal Microbiota Transplantation Combined With Biofeedback for Mixed Constipation: A Retrospective Cohort Study

**DOI:** 10.3389/fmed.2021.746990

**Published:** 2021-10-20

**Authors:** Bo Yang, Hongliang Tian, Chen Ye, Zhiliang Lin, Di Zhao, Chunlian Ma, Jiangman Zhao, Shouxin Wu, Rongfeng Jiang, Ning Li, Huanlong Qin, Qiyi Chen

**Affiliations:** ^1^Intestinal Microenvironment Treatment Center of General Surgery, Shanghai Tenth People's Hospital, Tenth People's Hospital of Tongji University, Shanghai, China; ^2^Shanghai Zhangjiang Institue of Medical Innovation, Shanghai Biotecan Pharmaceuticals Co., Ltd., Shanghai, China

**Keywords:** mixed constipation, fecal microbiota transplantation, biofeedback - BFB, spontaneous bowel movements, 16S rRNA gene sequencing, gut microbiota diversity

## Abstract

This study aims to assess the effectiveness and safety of fecal microbiota transplantation (FMT) combined with biofeedback for patients with mixed constipation. Patients who received biofeedback (biofeedback group, *n* = 40) and those who received FMT combined with biofeedback (FMT combination group, *n* = 45) were enrolled. Spontaneous bowel movements (SBMs) frequency, Bristol Stool Form Scale (BSFS), and Patient Assessment of Constipation Symptoms (PAC-SYM) score were analyzed to evaluate the effect of treatment. Gastrointestinal Quality of Life Index (GIQLI) scores of patients were used to assess the quality of life, and the safety of FMT combination therapy was evaluated by the presence of adverse events. The 16S rRNA gene sequencing was performed on the fecal samples of 12 donors, feces of 31 patients before and after receiving FMT combination treatment. Comparing the biofeedback group and the FMT combination group 1 month after the treatment, significant differences were observed in the mean value of SBM frequency, BSFS, and PAC-SYM scores, which were 2.15 ± 1.05 vs. 3.61 ± 0.89 (*p* = 0.0031), 2.1 ± 0.9 vs. 2.5 ± 1.2 (*p* = 0.008), and 2.4 ± 0.5 vs. 2.2 ± 0.6 (*p* = 0.0021), respectively. Meanwhile, FMT combination therapy had long-term beneficial effects according to the data collected at six months and 12 months after the treatment. With respect to the quality of life, GIQLI scores were higher in the FMT combination group (103.6 ± 15.1) compared with that in the biofeedback group (88.7 ± 10.1) one month after administration (*p* = 0.0042). In addition, there were no significant differences between the two groups in adverse events, including abdominal pain, diarrhea, dizziness, nausea, vomiting, and other side effects. Results of 16S rRNA gene sequencing showing some well-known probiotics had significantly increased after FMT combination treatment compared with pre-FMT samples, such as Prevotella and Bifidobacterium. Findings of this study suggested that FMT combined with biofeedback could be effective and safe for patients with mixed constipation.

## Background

Chronic constipation is a common gastrointestinal disease with an estimated prevalence of 5 to 20% worldwide based on the Rome IV criteria (1, 2). Chronic constipation is characterized by trouble defecating, reduced stool frequency, or perceived incomplete evacuation of bowel movements (3). In the light of the changes in gastrointestinal structure and function, constipation can be categorized into slow-transit constipation (STC), outlet obstructive constipation, and mixed constipation (STC and outlet obstructive constipation) (4). Evidence showed that most patients who suffered from STC had associated outlet obstruction, and would develop to mixed constipation as the disease progresses (5, 6). Fecal microbiota transplantation (FMT) has been proposed as a therapeutic approach for functional gastrointestinal disease, especially for recurrent *Clostridium difficile* infection (7, 8). Our previous studies suggested that FMT was effective and safe for STC (9), with 66.7 and 42.9% clinical improvement and remission rates at our hospital, respectively (10). Meanwhile, we found that FMT in combination with soluble dietary fiber (pectin) had both short-term and long-term efficacy in treating STC (11). In addition, biofeedback has been demonstrated as a safe and effective treatment for mixed constipation secondary to slow colonic transport (12), since biofeedback therapy works primarily through strengthening pelvic floor muscles (13). In addition, it was demonstrated that the FMT could improve the clinical phenotype of constipation by affecting gastrointestinal motility (14). Whether FMT combined with biofeedback could give more benefits for the patients with mixed constipation aroused our curiosity. This study aims to explore the clinical efficacy and safety of FMT combined with biofeedback in mixed constipation.

## Materials and Methods

### Patients and Data Collection

This single-center retrospective cohort study includes patients who were treated with biofeedback or FMT combined with biofeedback therapy for mixed constipation between June 2017 and June 2019 at the Tenth People's Hospital Affiliated to the Tongji University. The diagnosis of mixed constipation is based on Rome IV diagnostic criteria (15). Patients with mixed constipation were divided into two groups mainly based on their willingness. The clinicians did not intervene in the grouping of patients to minimize the selection bias. The flow diagram of this study is shown in Supplementary Figure 1.

Patients were assessed for inclusion according to the following criteria: (1) aged 18 to 70 years; (2) body mass index (BMI) 18 to 25 kg/m^2^. Patients were excluded if they met either of the following criteria: (1) patients lacking data on spontaneous bowel movements (SBMs) frequency, Bristol Stool Form Scale (BSFS), Patient Assessment of Constipation Symptoms (PAC-SYM) score, Gastrointestinal Quality of Life Index (GIQLI) score, and adverse events in the electronic medical record; (2) patients who had a history or evidence of the gastrointestinal organic diseases or metabolic or endocrine diseases.

Following the aforementioned inclusion and exclusion criteria, 40 patients in the biofeedback group and 45 patients in the FMT combination group were recruited in this study. The following data were retrospectively extracted from the electronic medical records of the patients: clinical characteristics (age, sex), underlying disease, and concomitant drugs.

Bristol Stool Form Scale (16) was used to measure the stool form. PAC-SYM questionnaire (17) and GIQLI (18) were applied to evaluate the constipation-related symptoms, and the quality of life of patients in the past two wk, respectively. PAC-SYM consists of 12 symptoms, which are grouped into the three subscales: stool, abdominal, and rectal symptoms. For each item, scores range from 0 (not at all) to 4 (all the time), with the higher scores indicating a worse symptom. The GIQLI was assessed using a 36-item questionnaire on the aspects of emotional, social, and physical states and also gastrointestinal health. The score for each item on the questionnaire is calculated using a 5-point Likert scale, ranging from 0 (the least desirable) to 4 (the most desirable), with a maximum score of 144. The reference value of GIQLI for healthy people is 125.8 ± 13.0.

This study was conducted with the approval from the Ethics Committee of the Tenth People's Hospital Affiliated to Tongji University and in accordance with the ethical principles for the medical research outlined in the Declaration of Helsinki 1964 as modified by the subsequent revisions.

### Donor Screening and Stool Processing

Potential donors were screened by strict criteria to minimize risks of disease transmissions, according to Evaluation criteria for FMT of the donor (T/SBIAORG 001-2020) issued by the Shanghai Biopharmaceutics Industry Association (19). In this study, 12 donors were enrolled through a series of questionnaires and medical examinations, including but not limited to the etiology detection of blood and stool, underlying disease, medication history, defecation status, psychological state, sleep quality, age, and BMI (10, 19). A total of 40 fresh feces were donated by the 12 donors, producing 40 batches of the fecal suspension, as we described previously (10). Quality control of the donated feces referenced the diagnostic criteria for the samples of facial microbiota transplantation (T/SBIAORG 002-2020) (20). Each donor's stool suspension provided the treatment for one to eight recipients. Each fresh feces underwent 16S rRNA gene sequencing.

### Treatment

The way of administration of FMT was described as a previous study (11). Briefly, vancomycin (500 mg, two times per day) was given orally for three consecutive days. On the last day of antibiotic treatment, bowel lavage with two liters of macrogol solution was applied. The next day, fecal suspension (100 ml, once per day) was infused within 10 min through a nasoduodenal tube. The infusion was performed for 6 consecutive days. Patients who were treated with biofeedback therapy learned gradually to eliminate the inadequate sphincter contraction guided by the therapist and by the visual and auditory feedback of the electromyographic activity of the external anal sphincter during simulated defecation (12). They received 20 treatment sessions (five per week) across one month. After the discharge, the Kegel exercise was performed twice a day for 30 min each time.

### Fecal DNA Extraction and 16S rRNA Gene Sequencing

Fresh feces samples were collected by sterile collection tubes before and two months after FMT, then stored at −80°C until further analysis. Fecal DNA was extracted using the QIAamp PowerFecal Pro DNA kit (Qiagen, Hilden, Germany) following the instructions. The quantity and quality of the DNAs were measured using the NanoDrop ND-1000 spectrophotometer (Thermo Fisher Scientific, Waltham, Massachusetts, USA), and agarose gel electrophoresis was performed. PCR amplification of the bacterial 16S rDNA genes V4 region was performed using the forward primer 515F (5'-GTGCCAGCMGCCGCGGTAA−3') and the reverse primer 806R (5'-GGACTACHVGGGTWTCTAAT-3'). The PCR reaction volumes were 50: 25 μl of Phusion High-Fidelity PCR Master Mix with HF Buffer, 3 μl (10 uM) of each forward and reverse primer, 10 μl of DNA template, and 6 μl of ddH_2_O. The reactions were set up to perform the PCR amplification using the following program: initial denaturation at −98°C for 30 sec, followed by 25 cycles consisting of denaturation at 98°C for 15 sec, annealing at 58°C for 15 sec, and extension at 72°C for 15 sec with a final extension at 72°C for one min. The PCR amplicons were purified using an AMPure XP Beads (Beckman Coulter, Indianapolis, IN) and quantified using the PicoGreen dsDNA Assay Kit (Invitrogen, Carlsbad, California, USA). After the quantification step, pair-end 2 × 150 bp sequencing was performed using the Illumina HiSeq4000 platform.

### Bioinformatics

After the quality control, the operational taxonomic unit (OTU) was analyzed using Vsearch-1.11, sequences were grouped into OTUs with a sequence identity similarity threshold of 97%. An OTU table was further generated to record the community composition of each sample at taxonomic ranks: kingdom, phylum, class, order, family, and genus. Sequence data analyses were mainly performed using the QIIME and R packages (v3.2.0). Use QIIME to calculate OTU-level alpha diversity indices such as Chao1, ACE, Shannon, and Simpson index. To compare the richness and evenness of OTUs among samples, OTU level ranked abundance curves were generated, dilution curves were drawn, and Alpha diversity index between groups analyses were carried out. Beta diversity analysis was performed by the QIMME to measure the UniFrac distance metrics and visualized *via* principal-component analysis (PCoA) to investigate the compositional differences among the microbial habitats across samples. To identify taxa with differing relative abundances between the two groups, linear discriminant analysis (LDA) effect size (LEfSe) analyses were performed. Microbial functions were predicted by Phylogenetic Investigation of Communities by Reconstruction of Unobserved States (PICRUSt).

### Outcomes

The primary outcome was the frequency of bowel movements per week. Secondary outcomes included the BSFS, PAC-SYM score, GIQLI score, and the presence of the main adverse events.

### Statistical Analysis

Statistical analysis was performed to compare bowel movement frequency, BSFS, PAC-SYM score, and GIQLI score between the FMT combined with biofeedback (FMT combination) group and biofeedback (biofeedback) group using *t*-test. To compare the presence of adverse events, Fisher's exact probability test was used. The significance level was set as *p* < 0.05.

## Results

### Baseline Characteristics of the Patients

A total of 40 patients administered with biofeedback and 45 patients administered with FMT combined with biofeedback were included in this study. There were no significant differences between the FMT combination group and the biofeedback group with respect to age, sex, and BMI (Table 1). The mean disease course of the FMT combination group and biofeedback group was 7.2 ± 3.1 and 7.4 ± 3.5 years, respectively. There were no significant differences between the groups in the aspect of complicated cardiovascular diseases, diabetes, or Parkinson's disease. Furthermore, there were no significant differences between the groups with respect to the periodical oral dosing of senna extract, bisacodyl, aole paidu capsule, and rhubarb used for the treatment of constipation. However, polyethylene glycol-electrolyte powder and phenolphthalein tablets were also used significantly more in the biofeedback group as compared with that used in the FMT combination group (*p* = 0.008 and *p* = 0.005, respectively, Table 1).

**Table 1:** Clinical characteristics of patients in Biofeedback groups and FMT combination group.

	**Biofeedback group (*n* = 40)**	**FMT combination group(*n* = 45)**	***p*-Value**
Age	45.5 ± 7.6	46.1 ± 7.1	>0.05
Sex (Male/Female)	14/26	14/31	>0.05
BMI (kg/m^2^)	21.5 ± 3.4	21.6 ± 2.7	>0.05
Underlying disease			
Diabetes	6 (15%)	8 (17.8%)	>0.05
Cardiovascular disease	5 (12.5%)	7 (15.6%)	>0.05
Parkinson's disease	2 (5%)	1 (2.2%)	>0.05
Concomitant laxative			
Senna extract	19 (47.5%)	23 (51.1%)	>0.05
Polyethylene glycol-electrolyte powder	29 (72.5%)	19 (42.2%)	**0.008**
Bisacodyl	11 (27.5%)	21 (46.7%)	>0.05
Aole paidu capsule	14 (40%)	11 (24.4%)	>0.05
Phenolphthalein tablets	15 (37.5%)	5 (11.1%)	**0.005**
Rhubarb	6 (15%)	10 (22.2%)	>0.05

### Effects of FMT Combined With Biofeedback

**Figure 1:**
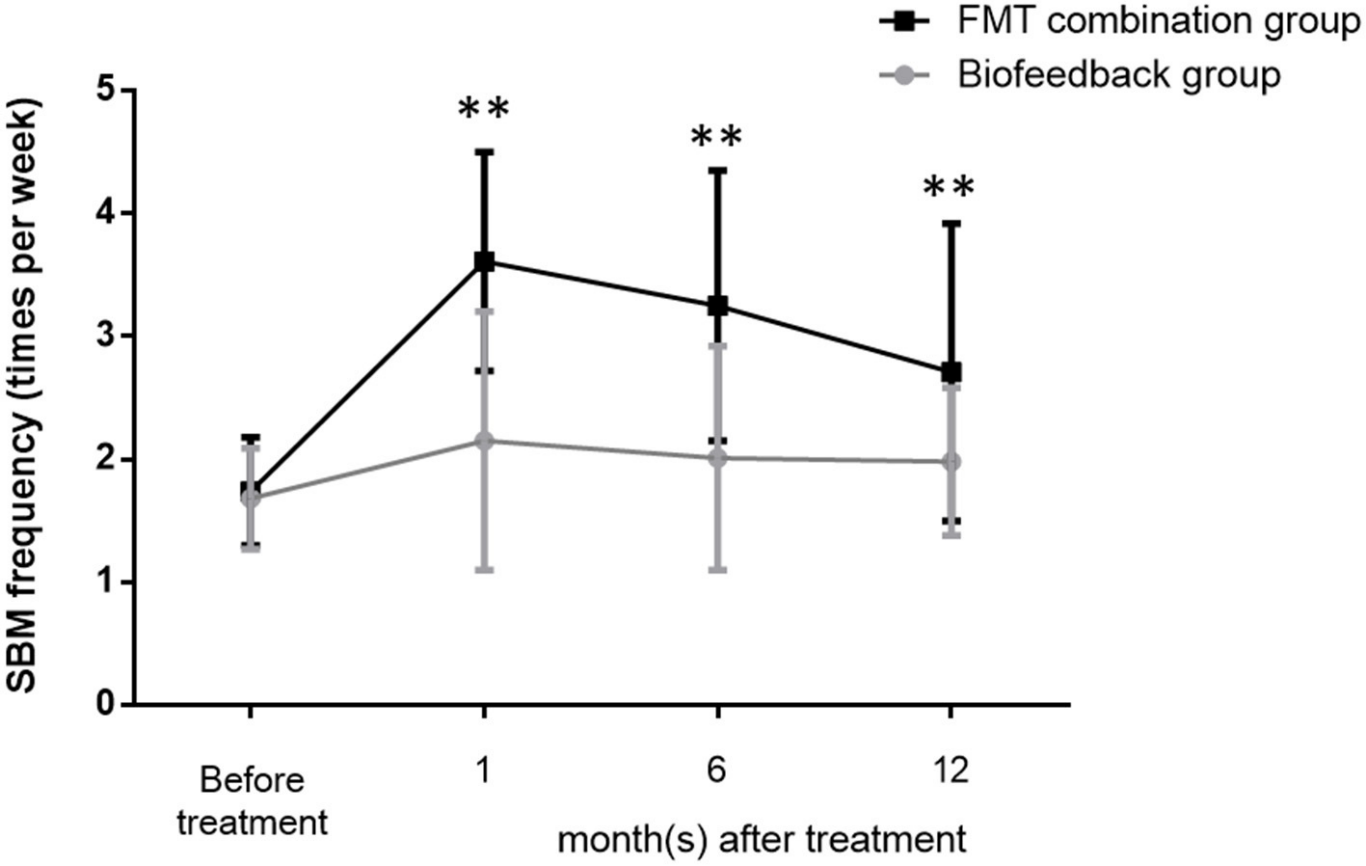
Changes in the frequency of bowel movement per week before and after treatment in the biofeedback group and the FMT combination group. The number of bowel movements per week before administration and up to 12 months after treatment is indicated by the mean ± SD. *p* < 0.01 according to the *t*-test are indicated by ^**^.

The mean values of SBMs frequency within one wk before treatment were 1.68 ± 0.41 and 1.74 ± 0.44 in the biofeedback group and the FMT combination group, respectively (*p* > 0.05). The frequency of SBMs (times per week) at one month after treatment were 2.15 ± 1.05 in the biofeedback group and 3.61 ± 0.89 in the FMT combination group, respectively, which were significantly different (*p* = 0.0031). Furthermore, the mean values of SBMs frequency at 6 and 12 months after treatment were 2.01 ± 0.91 and 1.98 ± 0.6 in the biofeedback group. In comparison, the frequency of SBMs 6 and 12 months after FMT combined with biofeedback was 3.25 ± 1.1 and 2.71 ± 1.21 times per week (*p* = 0.0024 and *p* = 0.0046, respectively, Figure 1). According to the exclusion criteria, the number of patients in the biofeedback group and the FMT combination group when 1/6/12 month(s) after the corresponding therapy were 37 and 38/32 and 36/16 and 25, respectively.

### Adverse Events in FMT Combined With Biofeedback

Table 2 describes the adverse events between the biofeedback group and the FMT combination group. The adverse events included abdominal pain, abdominal distension, anal pain, diarrhea, nausea, fever, palpitations, dizziness, allergies, vomiting, and chest tightness. The results showed that there was no significant difference in the adverse events between the two groups. Abdominal pain (7.5 vs. 15.6%, *p* = 0.209), abdominal distension (7.5 vs. 11.1%, *p* = 0.425), and anal pain (7.5 vs. 8.9, *p* = 0.567) were the Top 3 frequency adverse events both in the biofeedback groups and FMT combination groups. In addition, diarrhea (*n* = 4), fever (*n* = 3), allergies (*n* = 2), vomiting (*n* = 1), and chest tightness (*n* = 1) only appeared in the FMT combination groups, but not in the biofeedback group.

**Table 2:** Adverse Events after treatment in the Biofeedback group and FMT combination group.

	**Biofeedback group (*n* = 40)**	**FMT combination group (*n* = 45)**	***p*-Value**
Abdominal pain	3 (7.5%)	7 (15.6%)	0.209
Abdominal distension	3 (7.5%)	5 (11.1%)	0.425
Anal pain	3 (7.5%)	4 (8.9%)	0.567
Diarrhea	0 (0%)	4 (8.9%)	0.074
Nausea	1 (2.5%)	3 (6.7%)	0.354
Fever	0 (0%)	3 (6.7%)	0.144
Palpitations	1 (2.5%)	2 (4.4%)	0.545
Dizziness	1 (2.5%)	2 (4.4%)	0.545
Allergies	0 (0%)	2 (4.4%)	0.277
Vomiting	0 (0%)	1 (2.2%)	0.524
Chest tightness	0 (0%)	1 (2.2%)	0.524

### Secondary Outcomes of FMT Combined With the Biofeedback

In addition, we also analyzed the secondary outcomes data of patients, including BSFS, PAC-SYM score, GIQLI score at one, six, and 12 months after the treatment. The mean values of BSFS (average ± SD) before the treatment were 1.5 ± 0.5 and 1.6 ± 0.5 in the biofeedback group and the FMT combination group, respectively (*p*> > 0.05). One month after treatment, BSFS were 2.1 ± 0.9 in the biofeedback group and 2.5 ± 1.2 in the FMT combination group (*p* = 0.008). BSFS at 6 and 12 months after treatment were 1.9 ± 0.8 and 1.8 ± 0.5 in the biofeedback group, and 2.4 ± 0.9 and 2.3 ± 1.1 in the FMT combination group, which was significantly different (*p* = 0.0163 and *p* = 0.047, respectively, Table 3). The PAC-SYM score before the treatment were 2.8 ± 0.4 and 2.9 ± 0.5 in the biofeedback group and the FMT combination group, respectively (*p* > 0.05). The PAC-SYM score was 2.4 ± 0.5 in the biofeedback group and 2.2 ± 0.6 in the FMT combination group at one month after treatment (*p* = 0.0021). The PAC-SYM score at six and 12 months after the treatment were 2.6 ± 0.4 and 2.5 ± 0.4 in the biofeedback group, and 2.4 ± 0.5 and 2.3 ± 0.6 in the FMT combination group (*p* = 0.0023 and *p* = 0.001, respectively, Table 4). Combined with the results of SBMs, these results indicated that the FMT combination therapy had a better effect on mixed constipation as compared with the biofeedback treatment. With respect to the quality of life, GIQLI scores before the treatment were 80.5 ± 7.8 and 85.4 ± 13.2 in the biofeedback group and the FMT combination group, respectively (*p* > 0.05), while after the treatment, the GIQLI scores were higher in the FMT combination group as compared with that in the biofeedback group. As in Table 5, the GIQLI scores at 1 month were 88.7 ± 10.1 in the biofeedback group and 103.6 ± 15.1 in the FMT combination group (*p* = 0.0042), and GIQLI scores at 6 and 12 months were 86.2 ± 11.3 and 85.7 ± 10.8 in the biofeedback group, and 98.4 ± 13.2 and 95.6 ± 11.6 in the FMT combination group (*p* = 0.0035 and *p* = 0.0024, respectively).

**Table 3:** The comparison of Bristol score between Biofeedback group and FMT combination group before and after treatment.

	**Bristol stool form scale**			
	**Before administration**	**1 M**	**6 M**	**12 M**
Biofeedback group	1.5 ± 0.5	2.1 ± 0.9	1.9 ± 0.8	1.8 ± 0.5
FMT combination group	1.6 ± 0.5	2.5 ± 1.2	2.4 ± 0.9	2.3 ± 1.1
*p*-Value	>0.05	**0.008**	**0.0163**	**0.047**

**Table 4:** The comparison of PAC-SYM score between Biofeedback group and FMT combination group before and after treatment.

	**PAC-SYM score**			
	**Before administration**	**1 M**	**6 M**	**12 M**
Biofeedback group	2.8 ± 0.4	2.4 ± 0.5	2.6 ± 0.4	2.5 ± 0.4
FMT combination group	2.9 ± 0.5	2.2 ± 0.6	2.4 ± 0.5	2.3 ± 0.6
*p*-Value	>0.05	**0.0021**	**0.0023**	**0.001**

**Table 5:** The comparison of GIQLI score between Biofeedback group and FMT combination group before and after treatment.

	**GIQLI score**			
	**Before administration**	**1 M**	**6 M**	**12 M**
Biofeedback group	80.5 ± 7.8	88.7 ± 10.1	86.2 ± 11.3	85.7 ± 10.8
FMT combination group	85.4 ± 13.2	103.6 ± 15.1	98.4 ± 13.2	95.6 ± 11.6
*p*-Value	>0.05	**0.0042**	**0.0035**	**0.0024**

### Gut Microbiota Diversity Elevated of Patients Receiving FMT Combination Treatment

**Figure 2:**
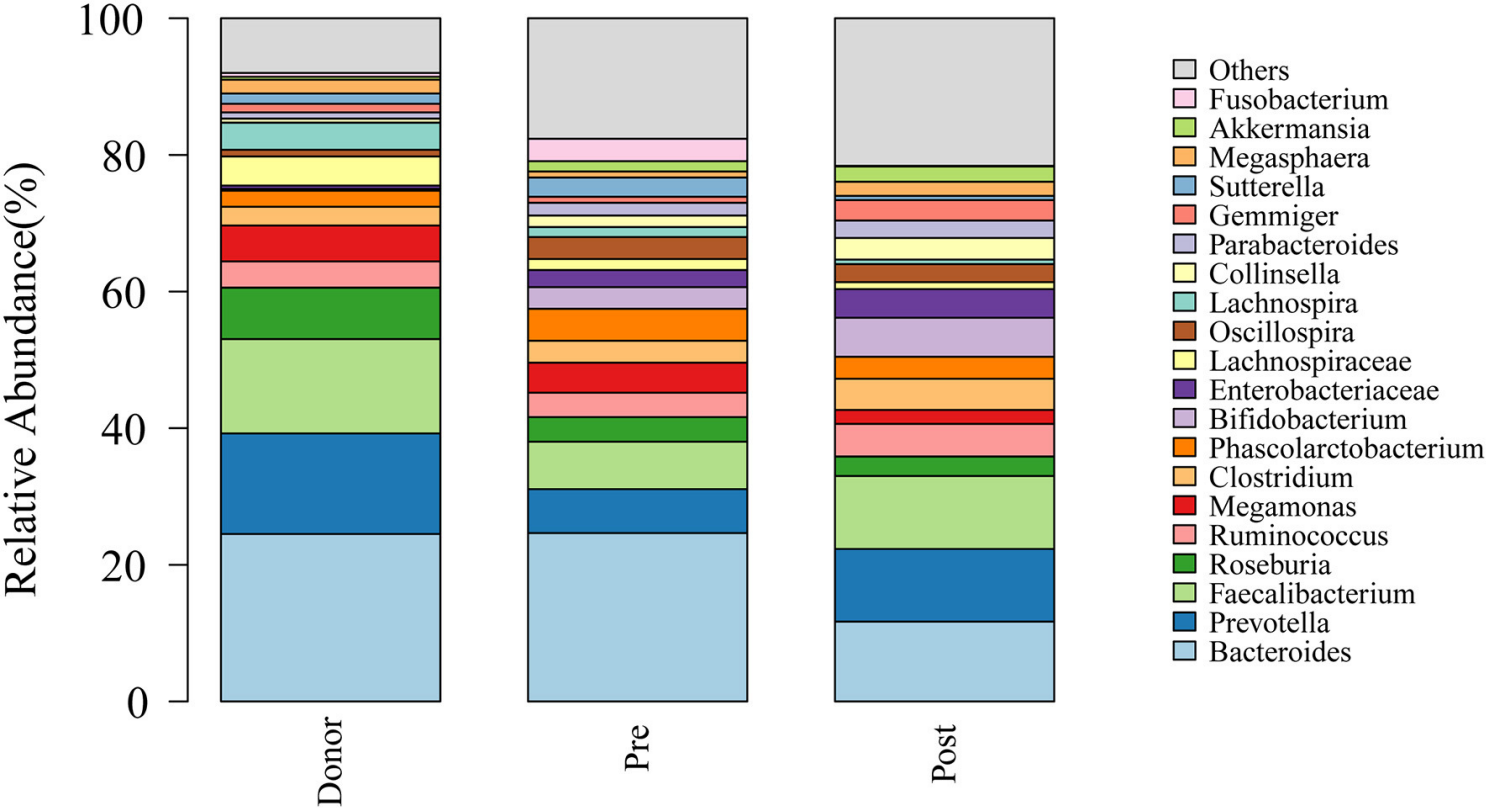
Genus distribution of the gut microbiomes of the donors, mixed constipated patients before and after receiving FMT combination therapy.

**Figure 3:**
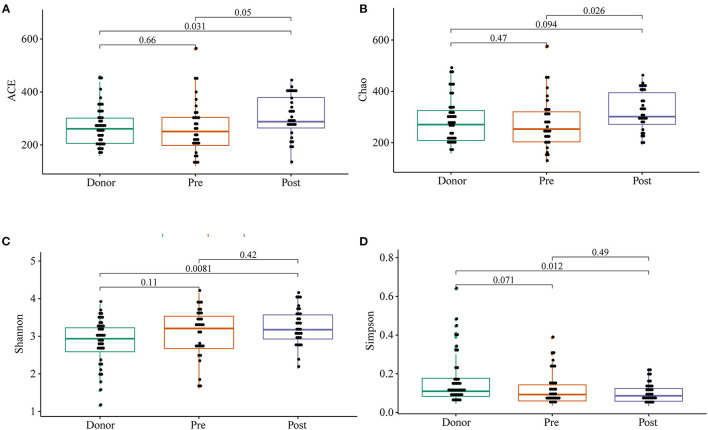
ACE **(A)**, Chao **(B)**, Shannon **(C)**, and Simpson **(D)** were used to analyze the alpha diversity of the gut microbiomes of the donors, as well as patients before and after receiving an FMT combination therapy. Statistical analysis used Wilcoxon rank-sum test.

**Figure 4:**
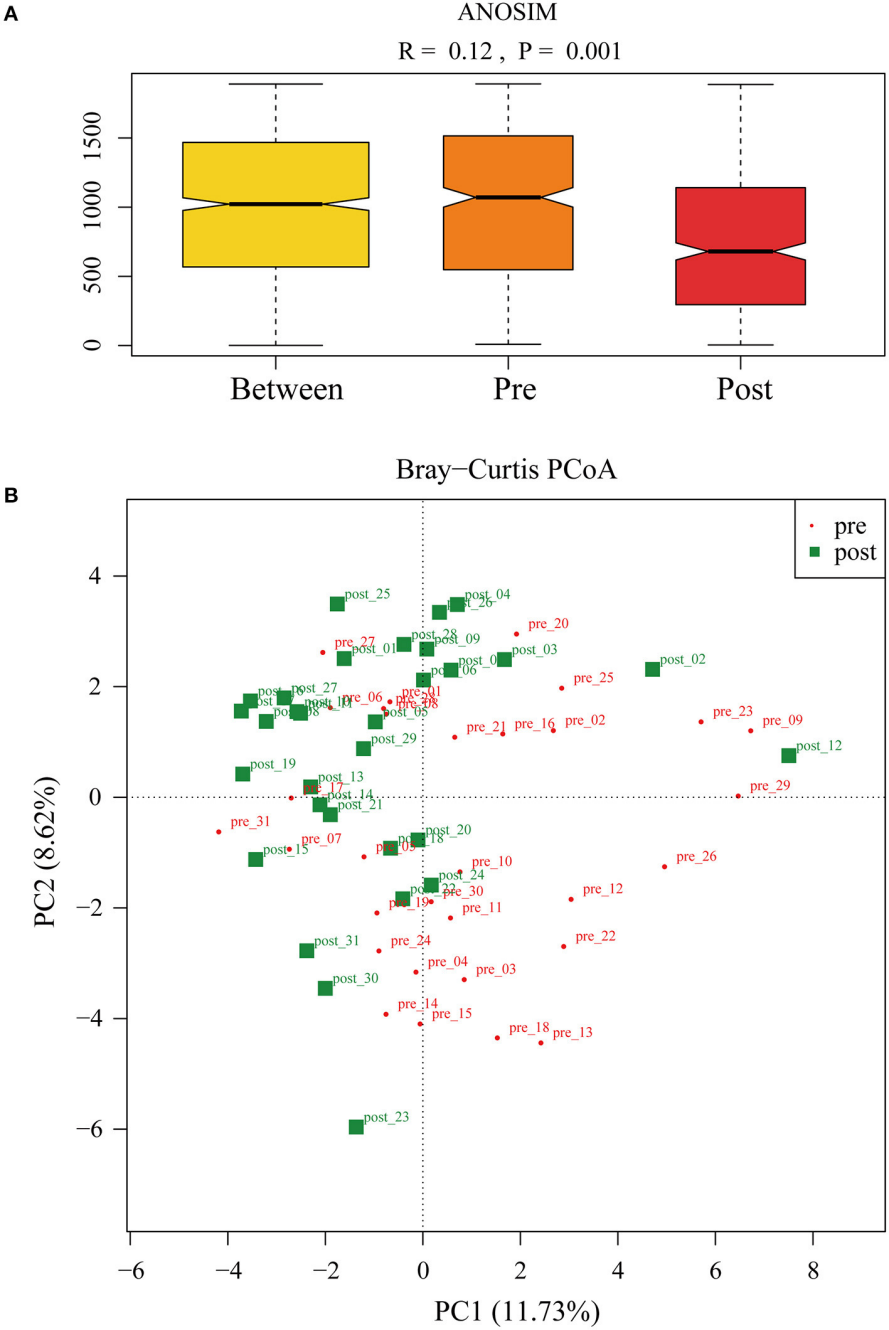
**(A)** ANalysis Of Similarities was used to compare the differences in each group and between the two groups (pre- and post-FMT) in the microbiota structure. **(B)** Principal-component analysis of the fecal microbiota between pre- and post-FMT groups.

To further investigate the role of the gut microbiota on the patients of FMT combination treatment, feces of the donor and feces of the patient before and 2 months after FMT underwent 16S rRNA gene sequencing. Figure 2 showed the microbiota composition at the genus level. Bacteroides, Prevotella, and Faecalibacterium were the major components in the feces of the donors (Figure 2). Figure 3 compared the alpha diversity (the ACE, Chao1, Shannon, and Simpson) of feces of the donors and pre- and post-FMT feces of the patients. We found that there were no significant differences on ACE (*p* = 0.66, Figure 3A), Chao (*p* = 0.47, Figure 3B), Shannon (*p* = 0.11, Figure 3C), and Simpson (*p* = 0.071, Figure 3D) index between the donors and patients with mixed constipation. After receiving FMT combination therapy, ACE (*p* = 0.05, Figure 3A) and Chao (*p* = 0.026, Figure 3B) were significantly higher in the post-FMT samples as compared with that in the pre-FMT samples. In addition, the patients after FMT combination therapy have a higher ACE (*p* = 0.031), Shannon (*p* = 0.0081), and lower Simpson (*p* = 0.012) as compared with the donors. For the beta diversity, principal-component analysis (PCoA) of a non-metric multidimensional scaling plot (on a Bray-Curtis distance matrix) and an unweighted UniFrac distance revealed significant qualitative differences in the gut microbial community structure before and after FMT combination therapy (Figure 4).

### Differences in Microbial Communities After FMT

**Figure 5:**
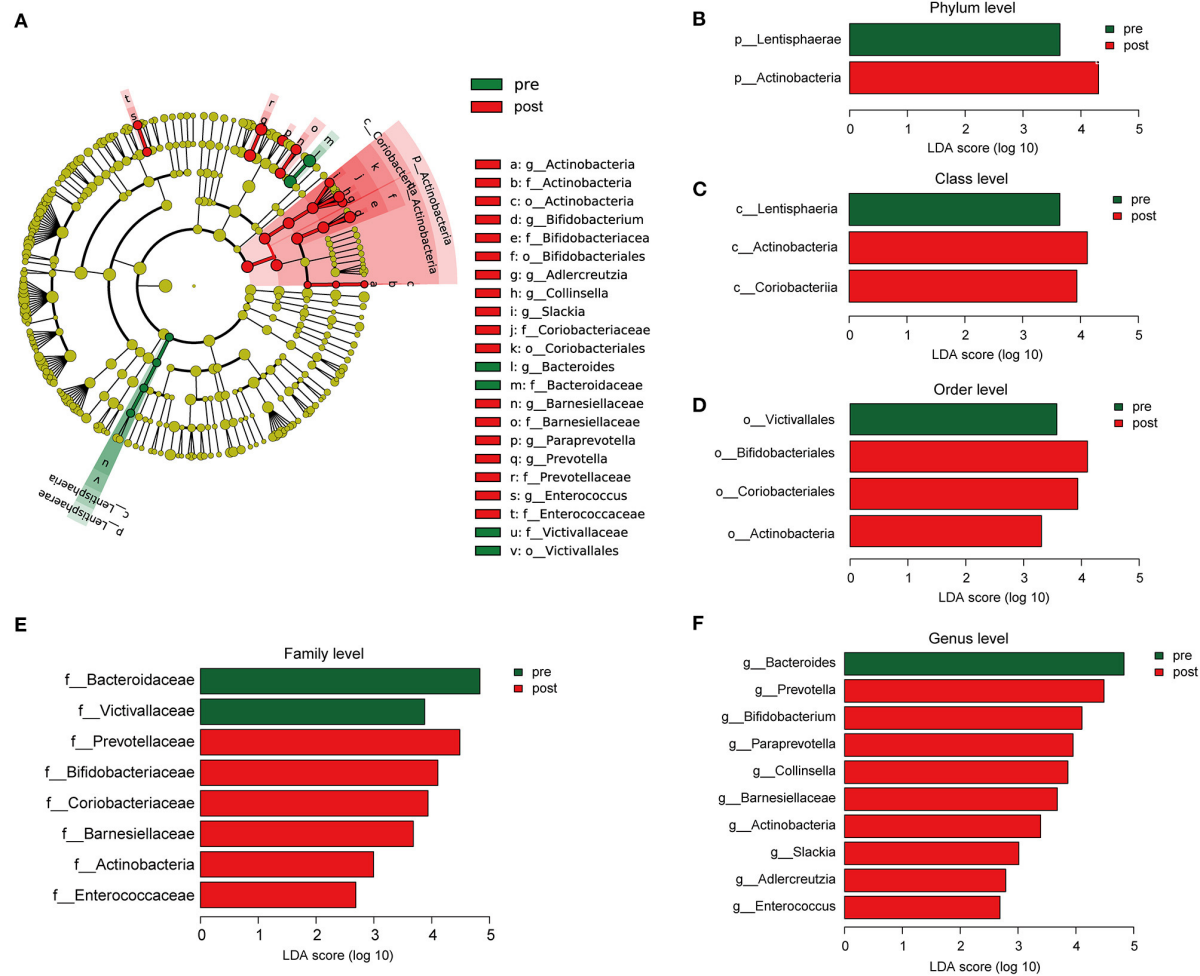
The different composition of the gut microbiota between the patients with pre- and post-FMT. **(A)** The cladogram depicts the phylogenetic distribution of microbial lineages in fecal samples taken before and after 2-month FMT combination treatment. **(B–F)** Key altered phylotypes of the gut microbiota after FMT treatment in phylum **(B)**, class **(C)**, order **(D)**, family **(E)**, and genus levels **(F)**.

Cladograms were plotted by the LEfSe analysis, which presented the most significant difference at the taxonomic levels between pre- and post-FMT groups (Figure 5A). Figures 5B–F, respectively, showed the high- and low-dimensional biomarkers at phylum, class, order, family, and genus levels in the patients with post-FMT gut microbiota as compared with that of the patients with pre-FMT. At the phylum level, the abundance of Actinobacteria significantly increased, while Lentisphaerae decreased (Figure 5B). At the class level, Actinobacteria, Coriobacteriia increased, while Lentisphaeria decreased (Figure 5C). At the order level, Bifidobacteriales, Coriobacteriales, and Actinobacteria increased, while Victivallales decreased (Figure 5D). At the family level, Prevotellaceae, Bifidobacteriaceae, Coriobacteriaceae, Barnesiellaceae, Actinobacteria, and Enterococcaceae increased, while Bacteroidaceae, Victivallaceae decreased (Figure 5E). At the genus level, Prevotella, Bifidobacterium, Paraprevotella, Collinsella, Barnesiellaceae, Actinobacteria, Slackia, Adlercreutzia, and Enterococcus increased, while Bacteroides decreased (Figure 5F).

**Figure 6:**
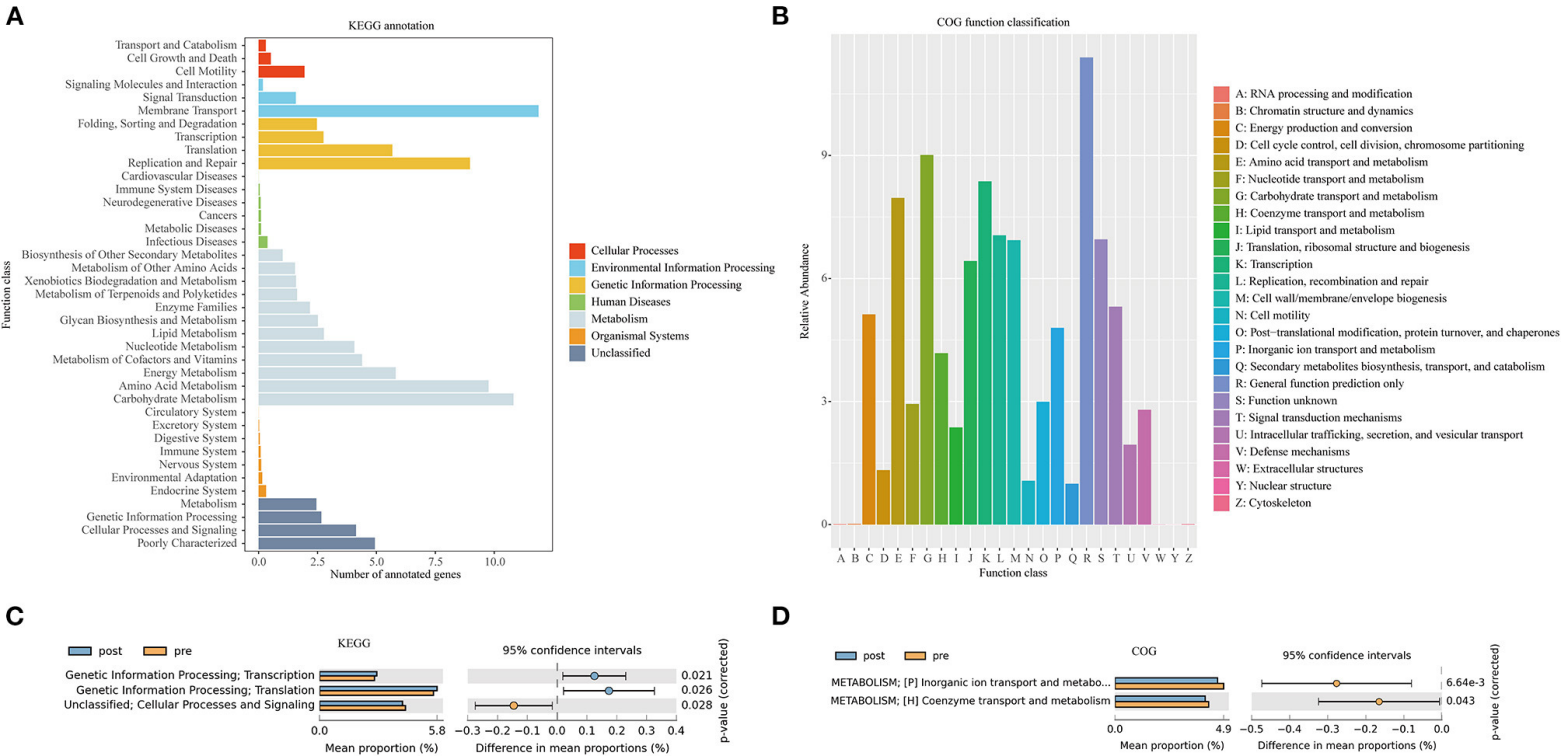
Kyoto Encyclopedia of Genes and Genomes (KEGG) annotations **(A)** and Clusters of Orthologous Genes (COG) function classification **(B)** by PICRUSt-predicted based on 16S rDNA gene sequencing data of the patients with mixed constipation. Differential KEGG pathways **(C)** and COG functions **(D)** in the patients with mixed constipation before and after 2-month FMT combination therapy.

Figure 6A presents the Kyoto Encyclopedia of Genes and Genomes (KEGG) annotation (Figure 6B) and Clusters of Orthologous Genes (COG) function (Figure 6B) predicted by PICRUSt based on the sequencing data of microbiome from all the patients. KEGG pathway (Figure 6C) and COG function (Figure 6D) in the second-level classification comparisons were performed to explore potential differences in the functional composition of the microbiome between pre- and post-FMT patients by the STAMP software based on the results of PICRUSt. Genetic information processing (transcription and translation) of the gut microbiota was more active after receiving the FMT combination therapy (Figure 6C). Inorganic ion transport and metabolism, coenzyme transport, and metabolism in the gut microbiota of the patients with post-FMT were not as active as that in the patients with pre-FMT.

## Discussion

Mixed constipation is a kind of chronic constipation with STC and outlet obstruction, difficult to treat in clinical practice (21). In this study, we aim to assess the efficacy and safety of FMT combined with biofeedback on mixed constipation. Based on the results in our study, FMT combination therapy possibly has a superior long-term effect over the biofeedback treatment.

Fecal microbiota transplantation refers to the transplantation of the functional flora from a healthy donor feces into the intestine of the recipient, which aims to restore the diversity of the intestinal flora and achieve the purpose of treating intestinal and extra-intestinal diseases. In the late 1980s, Borody et al. (22) performed FMT on four patients with chronic constipation and demonstrated a significant improvement in the defecation frequency, as well as immediate improvements in symptoms, such as abdominal pain, early satiety, and nausea after FMT. A prospective study reported that FMT might play its role through the restoration and colonization of the donor microbiota in the gut of the recipient up to one year after FMT (23). It has been considered that the FMT could be a promising choice for patients who are refractory to conventional therapeutic strategies.

In our previous randomized controlled trial (RCT) study, patients who underwent FMT had significantly improved symptoms, including fecal properties and defecation frequency, compared with the conventional treatment group (24). Meanwhile, biofeedback therapy has been demonstrated to be more effective with good long-term results as compared with sham therapy, laxatives, or the anti-anxiety drug diazepam for patients with constipation associated with a rectal evacuation disorder (25–27). In this study, we found that FMT combined with biofeedback had a sustained effect on mixed constipation. After one year of follow-up, patients who received FMT combination therapy showed relatively more frequent defecation, milder constipation-related symptoms, and better quality of life, compared with those who were treated with the biofeedback only.

Microorganism residing in the gut is believed to have a profound impact on the human physiology and nutrition, and are of the essence for human life. It has been found that the disruption of the homeostasis between the microbiota and the host has a more vital role as compared with the host genetics in the development of a range of diseases, such as inflammatory bowel disease, obesity, and type two diabetes (28). Increasing evidence uncovered that chronic constipation is associated with striking changes in the gut flora (29). Although without significance, we found that the patients with constipation had an increased quantity and diversity of bacteria as compared with the healthy donors, which has been reported in our previous study (14) and other researches (30). The study by Mattea Müller (31) had verified that slow distal colonic transit and hard stools are associated with an increased gut microbiota diversity. Although lack of clear consensus, patients with constipation have a lower abundance of Actinobacteria (32), including Bifidobacteria (32–34) and Prevotella (35) in their feces as compared with the healthy controls (29). Bifidobacterium is commonly used as a probiotic in adults (36, 37) and children (38) with constipation. In this study, the abundance of Prevotella, Bifidobacterium, and Actinobacteria have increased significantly in the guts of the patients after receiving FMT combination therapy. These results indicated that the FMT could remodel the gut microbiota composition of patients with mixed constipation, especially upregulate the beneficial bacterium that mainly explained the efficacy of FMT. Beyond that, some other significantly altered bacteria were also found. For example, Bacteroides was the most significantly downregulated community after an FMT combination therapy, but interestingly, healthy donors also have a relatively high abundance of Bacteroides (Figures 2, 5F). Previous studies give conflicting results of Bacteroides abundance, some reported higher (39, 40) and others reported lower (32) in the patients with constipation. We speculated Bacterioides have the highest abundance in gut microbiota that was susceptible to composition change of other bacteria but have slight effects on constipation.

Regarding safety, FMT has been generally considered a safe and well-tolerated treatment (41). In this study, there was little difference in adverse events between FMT combination groups and biofeedback groups. The adverse events in the FMT combination group were mostly short-term and mild, which were known to be associated with the delivery methods. We noticed that the low-grade fever appeared in three patients of the FMT combination group, but not in the biofeedback group. The appearance of fever is probably because of the result of a temporary systemic immune response to the transplanted bacteria, other researchers have also reported low-grade fever as the side effect of FMT (42). Besides, the long-term immunologic effects of FMT should also be concerned in the follow-up period after an FMT. Importantly, we should pay extra attention to the possible uncommon severe side effects following FMT, mainly referring to the risk of the infection transmission. Food and Drug Administration (FDA) have reported two cases of Extended Spectrum Beta Lactamase-producing *Escherichia* coli (*E*. coli) infection resulting in one death (43) and enteropathogenic *E*. coli in two cases and Shigatoxin-producing *E*. coli in four cases by donor stool (https://www.fda.gov/vaccines-blood-biologics/safety-availability-biologics/safety-alert-regarding-use-fecal-microbiota-transplantation-and-risk-serious-adverse-events-likely). Rigorous donor screening and testing could be mandated to minimize the risk of FMT. In recent years, management practices of FMT (44) and evaluation criteria for donors (19) have been issued in China, which were the safeguards of an FMT clinical application. Considering that mixed constipation is a kind of combination of STC and outlet obstructive constipation, which severely impacts the quality of life of those affected, findings of this study suggested that FMT combined with biofeedback would be beneficial to the patients relieving their symptoms and improving their quality of life.

The main limitation of this study is that it was in a retrospective setting. Strictly randomized control was difficult when patients were divided into two groups with a different therapy, which might cause selection bias. Meanwhile, there is a possibility that the diagnosis of chronic constipation and the indication of the treatment may vary among prescribing physicians. Thus, further cases are necessary for an in-depth evaluation. Since the mechanism of an FMT efficacy is comprehensive and 16S rRNA gene sequencing only explained it at the microbiome level, metabolome should be applied to the further probe into an FMT mechanism in the future study. At last, each donor provided stool suspension for one to eight patients, and the patients showed various degrees of efficacy. So, it was difficult to define the more effective donors.

In conclusion, FMT combined with biofeedback showed better effects and equal adverse events on mixed constipation, compared with biofeedback treatment. It also alleviated the constipation symptoms and improved the quality of life of the patients. However, a large-scale prospective study is still to be required to further assess the benefits and risks of an FMT combination therapy for mixed constipation.

## Data Availability Statement

The original contributions presented in the study are publicly available. This data can be found here: https://www.ncbi.nlm.nih.gov/bioproject/750874 (BioProject ID: PRJNA750874).

## Ethics Statement

The studies involving human participants were reviewed and approved by Ethics Committee of Shanghai 10th People's Hospital. The patients/participants provided their written informed consent to participate in this study.

## Author Contributions

QC and BY have made substantial contribution to the conception or design of the work. HT, DZ, CY, ZL, RJ, CM, and BY have made the acquisition, analysis, or interpretation of data for the work. NL, HQ, JZ, and SW have drafted the work or revised it critically for important intellectual content. BY have approved the final version to be published. BY agree to be accountable for all aspects of the work in ensuring the questions related to the accuracy or integrity of any part of the work are appropriately investigated and resolved. All authors contributed to the article and approved the submitted version.

## Funding

This study was supported by National Natural Science Foundation of China (No. 8210031248), Clinical Research Plan of SHDC (No. SHDC2020CR4026), Clinical Research Plan of SHDC (No. SHDC2020CR1030B), and the Shanghai Strategic New Development Project (BT-FGW-2019-06).

## Conflict of Interest

Authors Jiangman Zhao, Shouxin Wu and Rongfeng Jiang were employed by company Shanghai Biotecan Pharmaceuticals Co., Ltd. The remaining authors declare that the research was conducted in the absence of any commercial or financial relationships that could be construed as a potential conflict of interest.

## Publisher's Note

All claims expressed in this article are solely those of the authors and do not necessarily represent those of their affiliated organizations, or those of the publisher, the editors and the reviewers. Any product that may be evaluated in this article, or claim that may be made by its manufacturer, is not guaranteed or endorsed by the publisher.

## Supplementary Material

Supplementary Figure 1 Flow diagram of this study.
