# Supplementary figures and images for: The Efficacy and Safety of Fecal Microbiota Transplantation Combined With Biofeedback for Mixed Constipation: A Retrospective Cohort Study
Source: Front Med (Lausanne). 2021 Oct 20;8:746990. doi: 10.3389/fmed.2021.746990 (PMC8564017; doi:10.3389/fmed.2021.746990)

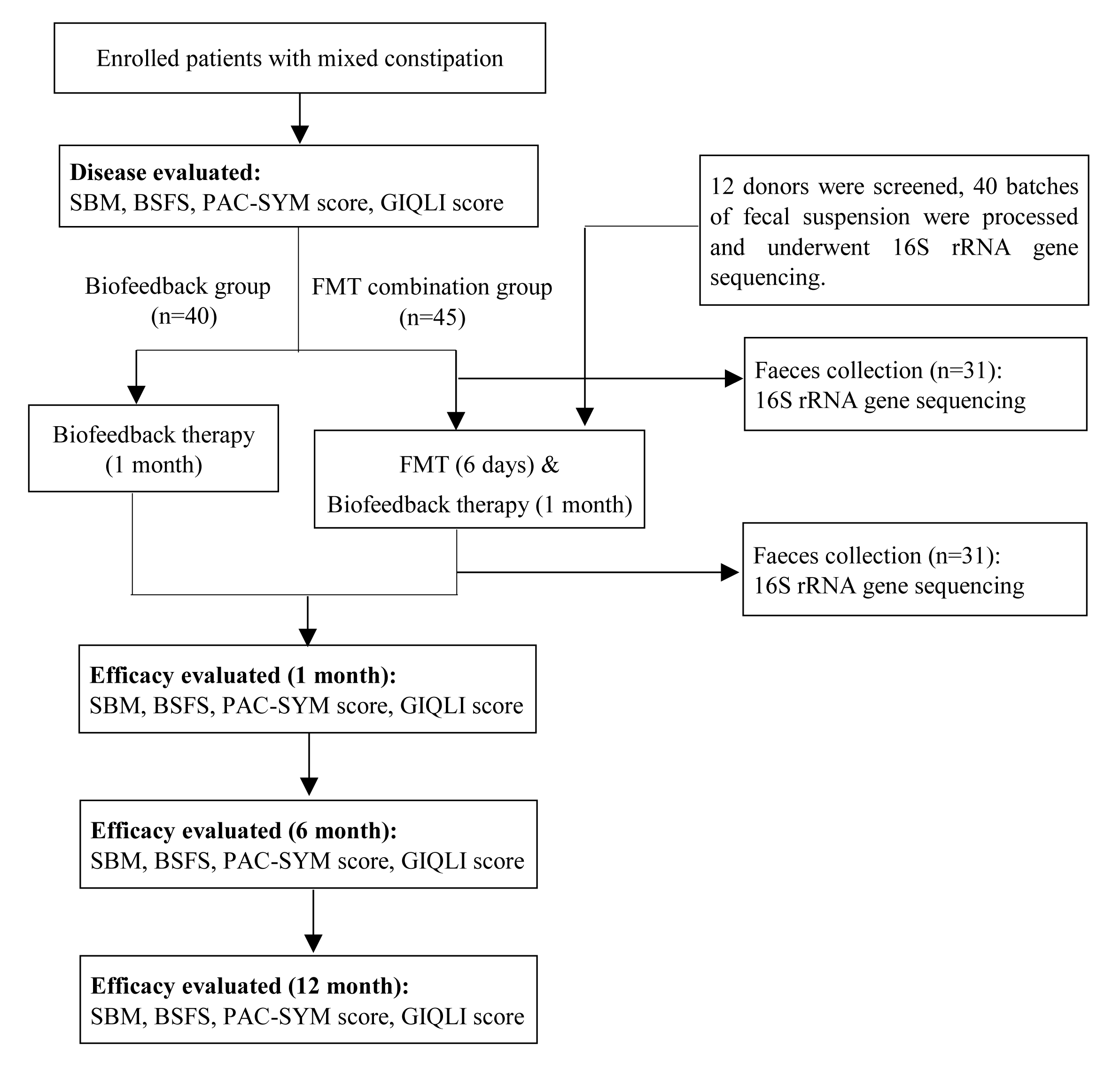

Supplement: Supplementary Figure 1 — Flow diagram of this study. [file Image_1.TIF]
